# Public healthcare disparities in Africa: the food production systems and its dichotomy in a South African context

**DOI:** 10.1186/s41043-023-00490-3

**Published:** 2024-06-01

**Authors:** M. G. Manamela, M. E. Choung

**Affiliations:** 1 Department of Development Studies, College of Human Sciences, University of South Africa, Pretoria, South Africa; 2 Department Communication, Media and Information Studies, School of Languages and Communications, University of Limpopo, Polokwane, South Africa

**Keywords:** Public healthcare, Organic food production system, Conventional food production system, Disparities, South Africa

## Abstract

One of the major concerns of development in Africa is the issue of public health. In Africa, public healthcare has been and still is a problem most African countries are faced with. The problem of public healthcare seems to be unabated even though there are measures that are put in place for its effectiveness. There is hunger, malnutrition, high mortality rate, illnesses and deterioration of life expectancy in most developing countries of Africa. The dramatic unprecedented public health disparity has become a scourge in developing countries where it has purportedly impaired the developmental efforts, economic growth and prosperity. As a result, there is a need to scrutinize possible causes that exacerbates public health issues in developing countries. The paper argues that the current food production system (conventional) contributes to current status of public health as compared to the previous food production system (organic). The purpose of this paper is to conceptualize public healthcare disparities, juxtaposing organic and conventional food production that result as human food consumption. The paper employs literature-based analysis as a methodology to assemble data in respect of public healthcare disparities and food production systems.

## Introduction

The paper provides the discourse of public healthcare disparities in Africa on the basis of the kinds of food production systems considered. That is without overlooking other public healthcare inequality when it comes to access to healthcare services which include medical aid cover. The issue of public healthcare is a major focus for most of developing countries in Africa [25]. Then, dealing with health equity in developing countries means striving for the possible equal level of access to healthcare services for all [11]. Concurrently, inequality in respect of access to healthcare services radiates the public health disparities considering both medical aid cover and food production systems in Africa. As a result, such situation determines the public healthcare disparities and related issues in Africa,considering the sprouting of various diseases, high mortality rate and deterioration of life expectancy. Accordingly, there are issues of starvation, hunger, malnutrition, Vitamin A deficiency, among others, in Africa that are unabated and escalating in the health system [13].

There are dramatic unprecedented public health disparities that have resulted as a scourge in developing countries where they have purportedly impaired the development efforts, growth and its prosperity since health become some major focus aspects in the twenty-first century [2]. As a result, there is a need to scrutinize what could be the causes that exacerbates public health issues in developing countries, particularly in the modern era. One of the main reasons of public healthcare disparities in Africa is medical aid cover access inequality [3, 35]. The status of public health issues and food production systems, respectively, are the main focal drive of the purpose of this paper. But that does not neglect the issue of equal access to medical aid cover for health by the public which exposes the public health disparities as well. Statistics South Africa (Stats SA) [33] reveals that, on one hand, access to medical aid is determined by the wealth while on the other hand approximately 70% of households use public health facilities. Furthermore, the 2017 General Household Survey (GHS) reveals that less than 17% in South African households possess medical aid for their health. Thus, the paper does not overlook the fact that there is a public health disparity in terms of medical aid, benefits and equal access to health services. Nonetheless, the paper posits that community food production systems play a pivotal role in alleviating public health issues in the modern era.

It can then be argued that modern/conventional food production system exacerbates public health issues due to its negative effects on human bodies. The paper is amenable with the notion that too much consumption of food produced through conventional food system causes emerging illnesses on human body, predominantly in developing countries. As a result, this can possibly affect the majority of people in developing countries negatively, since they are the ones who are experiencing public health disparities. Therefore, the public becomes vulnerable as result of conventionally produced products that have the potential of reducing life expectancy and escalating death rates because of malnutrition and other probable diseases [39]. Then public health system is affected due to the aforementioned rationale.

Conceptually, the main focus of this paper is on the implications and effects of food production systems that play a contributory role in public health issues. The questions can be on the basis of whether the food production, through the kind of systems employed, is good toward human bodies or not; while taking into cognizance the community food production systems which are deemed obsolete in the modern era. Accordingly, community food production systems can be classified as communal, household and school food production systems in that regard. Communal, household and school food production systems are regarded as organic farming whiles the modern food production as conventional farming. Thus, the food production systems should be considered as role players in ensuring food security beyond individual, household and community levels in terms of nutrients and health purposes.

The paper conceptualizes the two types that are considered for food production, particularly on health benefits. This is mainly because food production systems play a pivotal role when it comes to dealing with the dire and unabated public health issues in developing countries. Food production systems can either lead to the alleviation of and or contribute to various diseases with respect to public health issues. Moreover, the paper argues that there is a food strategy, which is community food production, that can ensure food security on the basis of nutritional aspects that can possibly lead to good human health. The paper also argues that conventional food production system does not serve as the optimum strategy in addressing hunger, malnutrition, high mortality rate and public health issues. The previous views are informed by an analyses on the benefits and capability of community food production system in respect of public health.

Within that context, the paper explores public healthcare issues on the basis of the transition of food production systems from organic to a contemporary one. Thus, the paper provides a soliloquy (a written discourse) on the basis of research methodology that details the gist and rationale as a supporting structure of the argument thereof. The premise and argument of the paper is driven by the previous (obsolete) and current food production systems that are perceived to be major contributors to the status of public health, particularly in developing countries. The paper conceptualizes public health disparities on the basis of juxtaposing organic and conventional food productions that result as human food consumption. In light of that, the paper’s framework is about the issues of public healthcare disparities and the dichotomy of food production systems.

## The juxtaposition and dichotomy of food production systems

The transition of organic food production system to the contemporary one provides a dichotomy on the issues of public healthcare and disparities. The complex nature of food production systems in the modern era contributes much in the public health issues [18]. Food production and consumption are major aspects to be considered when looking at the impacts of various aspects in relation to public health, social cohesion among others [27]. Accordingly, the crux of the juxtaposition and dichotomy is driven by the previous and current agricultural food production systems as well as the status of food and health in developing countries. Looking at the current/modern food production system, in the midst of agricultural technology, relative majority of people in developing countries are faced with hunger, malnourishment, diseases and environmental degradation [13, 18, 19, 29].

Evidently, too much food is produced to feed people well in the modern agricultural technology; conversely, people are well-fed but there is increasing concerns of unwell and public health issues in developing countries [18, 19]. Since the introduction of green revolution in developing countries there has been a plethora of health issues as well as challenges emanated due to its effects [18]. The challenges encapsulate the child mortality and fertility rate, reduction of life expectancy, high unemployment rate among others [13], World Health Organization [39]. According to Cantrell and Hettel [6], green revolution proved that poverty and hunger could be alleviated through the application of modern science and technology; without it, the number of poor and hungry today would be far greater. However, not disputing the contributions of green revolution in alleviating poverty and hunger, one could argue that poverty and hunger are still unabated in the presence of modern science and technology. Conversely, the introduction of green revolution affected the employment rate negatively due to modern science and technology. As a result, the possibility of high rate of poverty will enhance where the poor remaining poor and the rich becoming richer.

Thus, the problem of public healthcare disparities became a major focus to be dealt with, particularly in developing countries [8]. Subsequently, pursuing public health equity means striving for the highest possible standard of healthcare for all people and giving special attention to the needs of those at a greatest risk of poor health [8, 11]. For that reason, the importance of organic farming is highlighted as a crop biodiversity that is a key role player in helping farmers to improve their livelihoods while protecting the environment and families’ health [5, 8]. Interestingly, planting of traditional rice varieties alongside the green revolution seeds plant is to control pests and diseases on the products planted [18]. The previous statement highlights a key aspect and the pivotal role of organic farming in respect of health disparities and pests’ control. As a result, one can argue that organic farming has the capability to reduce negative effects of health toward a human body.

Most notably, relative majority of African countries rely on agriculture for the production of food and consumption [5, 32]. Through the ages, African countries have relied on traditional agriculture (organic farming) that has usually followed ecological wisdom unlike green revolution that has relied on technologies [5]. Thus, in contrast, green revolution produced products that lead to a change in dietary habits as many people are affected by malnutrition, in that regard, considering iron or vitamin-A deficiencies among others [18, 19, 39]. Meanwhile, organic farming is perceived as a way of yielding food products that can offer people benefits of direct access to nutritional food. Looking at green revolution production, the combination of pesticides and fertilizers that are assumed to increase food production has adverse effects on human health and environment [18, 21, 39]. As a result, this can possibly affect the health of many people mostly in the developing countries of Africa. Within that context, the issue of health, production and consumption call for a symbiosis with the ecology, cultural and social systems.

In line with the aforementioned, there is an overlook of community food production systems that could be helpful in eliminating the scourge of public health issues. The emphasis of community food production model is drawn from the fact that through its production, the human immune system of consumers may remain stronger and resist diseases and illnesses [23]. Hertel, Elouafi, Tanticharoen and Ewert [16] perceives the concept of community food production system as maintained by people who retain knowledge on the land and food resources rooted in historical continuity within their region of residence. It includes household food production system that is encapsulated within the traditional knowledge, considering natural environment through farming, particularly practicing organic farming and home gardening [16].

## Issues of public healthcare disparities in South Africa

It is worth noting that public health challenges have been and still are a global problem; that amounts to huge social and economic costs [3, 9, 15]. Most developing countries are faced with public health challenges which include undernutrition, overweight/obesity and/or micronutrient deficiencies [10]. In the modern era public healthcare is oriented more to the enrichment of doctors, pharmaceutical companies, hospitals and insurance companies than it is to actually taking care of self-reliant thereof [34]. Public healthcare disparities have been a challenge in most of the developing countries such as South Africa [2] and [35]. Statistics South Africa (Stats SA) [33] reveals that people who are able to access quality healthcare services are the middle class because they have access to medical aid, whereas approximately 70% of disadvantaged households use public healthcare services. The 2017 General Household Survey (GHS) revealed that less than 17% in South African households possess medical aid for their health. Within that context, the needs for public healthcare is escalating and amounting to exorbitant access to healthcare services. Accordingly, Betts and Korenda [4] suggests that if hospitals and other healthcare industries were owned by the people in the communities they serve, they would presumably become more interested in cheap remedies that actually work.

Concomitantly, in the twenty-first century, technologies are portrayed to be a way that gives people hope in fast tracking the production of food, but while blatantly overlooking issues of health. Ironically, it is recorded that the immune system of the public become weaker and the human body can easily be affected by diseases considering modern ways of food production [17]. Meanwhile, Avitsur et al. [1] confirmed that the weaker immune system is inept to resist various diseases that are emanating in the modern era, particularly on the maternal mortality that is evidently increasing in most developing countries. On that note, the weak human immune system leads to a backlog in the modern era to demand much care in respect of public health, particularly in the developing countries.

Looking at the modern/conventional food production and its negative effects on human health, one could juxtapose it with the benefits of community food production systems for health purposes. As a result, both the food production systems have a major role on the issues revolving around public healthcare. The interactions between human and food production lead to preparation and consumption of food underpinning health benefits and food security [28]. In light of that, a rate of food insecurity and obesity, as a health problem, has risen and is still rising due to the kind of food consumed including other effects in that regard [27]. Food insecurity is regarded as a major public health issue in Canada because of high rates of poverty, effects of climate change and environmental pollution on food systems [26]. According to the World Bank [38], poverty is among the greatest health hazards,thus, the community food production can aid in the reduction of poverty as a health hazard. The other problem could be the after effects of medication applied, notwithstanding the consumption of Genetically Modified Foods (GMFs), which possibly contribute to reduction of life expectancies. That could be the result of the modern/conventional food production systems. Therefore, it is increasingly significant to ensure that the issue of public healthcare is put in the hands of the affected people to better be combated than relying on healthcare industries. It means that household food production has the capability of reducing both poverty and the elements in relation to health issues. The reason why community food production is regarded as a path to combat issues of public health is the fact that it is resilient in an unpredictable climate and pest resistance.

## Perceptions around safety of food production

In numerous developing countries, food safety and awareness are acknowledged as the most important elements in relation to public healthcare, food security and poverty [14]. In most rural areas, there are dire effects of diseases resulting from poverty and vulnerability of food consumption [25, 27]. The result of various diseases and illnesses are assumed to be caused by conventional food production systems that utilize advanced food production technologies. As such, the concept of food technology has been adopted with the aim utilizing of advanced technologies for safety of food to be consumed as well as the side effects which may be experienced as a result of conventional food consumption [16].

Accordingly, there is a contestation about the health implications after the use of pesticides and herbicides amongst various scholars. For point of reference, Manamela and Molapo [18] and [22] posit that the use of pesticides and herbicides result in various health issues (such as endocrine disorders, cardiovascular diseases, negative effects on the male reproductive system, dementia, etc. in developing countries. For instance, agriculture is globally responsible for about a quarter of all greenhouse gas emissions as it occupies an estimated 40% of the earth’s surface [36]. This means 70% of freshwater resources and the excessive use of fertilizers increases the pollution on surface water used for irrigation of food products [36]. Hence, a relative majority of consumers in the modern era turn to be more reluctant to consume food produced through conventional food systems (Human Sciences Research Council 2004). That is because it makes people more concerned about the quality of food to be consumed and the ambience in which the food is produced.

On one hand, a trust factor in respect of food production systems plays a pivotal role toward individual’s decision on consumption due to uncertainty regarding the quality of food [31]. On the other hand, the environmental impacts of food production have also shown to be formidable for food consumption. This has led to a relative number of individuals rethinking their decision of consuming conventional food products. A study conducted by Hamed and Mohammed [14] paints a picture of just how individuals are finding it difficult to acknowledge the prominence and safety of conventional food products. Hamed and Mohammed [14] analyzed data that was collected through focus group discussion and questionnaire survey; it was observed that participants do not trust the safety of food produced through conventional food system and felt discouraged from consuming them. However, a relative number of consumers attested to have consumed fresh food not aware that certain or adulterated food borne bacteria can cause diseases that may lead to dreadful consequences including death [14]. It was then concluded that there is a need for individuals to know more about food safety and to stimulate positive attitude toward the safety of food consumption.

Those with mistrust of the locally produced food find themselves opting for the products sold at “reliable” markets, but there are those who may wish to do the same and not be able to. The income differences determine the purchasing activities which may take place in a situation where individuals do not trust the food source. Various studies have shown income disparities to have an influence on the perceptions of food safety and have detailed the perceptions about the safety of locally produced food [24, 40]. In addition, Messer, Constanigro, and Kaiser [20] note that there are numerous things that may make people skeptical about consuming conventional foods, however, in some instances consumers are forced to make a choice that disregards the safety of food they consume as a result of limited capital and nutrition knowledge.

## Benefits of community food productions on public health

There are three community food production systems that are the focal point of this paper which are communal, school and household food production systems. Concomitantly, these food production systems are regarded as an alternative, local and sustainable food systems that are designed to produce organic and healthy food to meet food needs while maintaining healthy ecosystems and combating diet-related diseases [12]. One could explicably validate the notion that community food production systems could be a viable strategy to deal with the continuous and unabated public health issues. In other countries, for case in point in America, the American Public Health Association considers community food systems as the one that provides healthy food to meet food dietary needs while maintaining healthy ecosystems [12]. While, the American Planning Association perceive community food production systems as designed to combat illness and or diseases such as obesity for the sake of public healthcare [12].

Consequently, organic food production system such as community food systems falls within other methods of crop production that relies much more on choosing not to use pesticides, fertilizers, genetically modified organisms, antibiotics among others [18]. This in fact has an influence on the type of food which people will choose to consume as there are those who are more health conscious and strict about what they consume. When more discrepancies are suspected, many may opt for organic foods that are nutritious and not genetically modified. Additionally, they may not use conventional fertilizers such as coal ash, which contains heavy toxic metals [7], to grow food as they may contaminate food plants to be produced due to chemicals active in pesticides and herbicides.

Within that context, community food production systems can be found in variety of models that encapsulate communal, school and household food production systems [37]. According to Shepon et al. [29], community food production system may mean many things to many people,to some is a place to grow food and whereas to some is a place to reconnect with nature or for physical exercises. These various models of food production systems may serve different purposes such as to reconnect with nature, physical exercise, demonstration, providing education and for selling or consuming produced food products [30]. School food production systems can also demonstrate and serve a purpose in education, although not overlooking others in that regard. On the basis of other purposes that exclude selling, produced food products can be handed to community members, more particularly child headed families and orphans for consumption. As a result, it may mean that community food production is either a place for growing food or for people practicing it for the purpose of physical exercise and educational purposes. Thus, consumption of organic produced nutrients foods can contribute to a better health of the public considering their benefits thereof.

## Discussion and recommendations

Although there seem to be progress with respect to the conventional food production system, improving and maintaining a good health status is of a concern. In line with development, food security and health may be improved by converging both the advantages of conventional and organic food production systems in developing nations. Additionally, the agriculturalization as a tenet of the engine growth in development can possibly increase safe food production and eliminate other health risks and threats [1, 12]. It can further be argued that not only will the process of agriculturalization enhance the lives of consumers, it will also ensure the social and economic development. With respect to agriculturalization, the conventional food production system seems to have a competitive edge over the organic food production system in the market related spectrum because it adequately responds to the ever-growing global population demand, thereby ensuring food security [32]. It is arguable that the organic food production system provides better access to health than the conventional food production system. Thus, Benatar et al. [3] and Dimitri [11] substantiate the argument by reflecting that health equity in developing countries is important because of striving for a possible level of access to healthcare.

The paper recommends that health policies should integrated with local and organic food production. To draw the focus on the integration of public efforts to ensure that people do not likely fall into severe health risks. Thus, the consumption of certain types of food plays a pivotal role in that regard hence community and household food production become pivotal for public health. There is a need to acknowledge and envision that community and household food production is an alternative that is needed to work for a change in the public health system and related issues thereof. Strategies to be considered are mini-stands and gardening in both rural and urban areas. These unique food strategies can ensure a good harvesting, sharing and consumption that are cost-effective. Furthermore, the strategies can impact the four pillars of food security which are access, availability, utilization and stability. There is a need of resources for health purposes that include not only medical care facilities but also health-promotion through community and household food production; and consumption of food for the public. This may be important for regulatory agencies, policy makers to consider and perhaps subsidize prices for quality food to be accessible to all consumers and create awareness about safety and risk of purchasing potentially contaminated foods as that may enable consumers to make informed decisions when purchasing food. All the process of community and household food production systems for the purpose of public health can be done through:

- Supervision and controlling from the Department of health, monitoring corporation, and other relevant institutions concerning food safety management,
- Conducting more relevant research about the benefits of community and household food production systems,
- Creating awareness campaigns for residents in marginalized areas who lack knowledge about nutrition information for safety consumption. This can be done by the Department of Health in partnership with the food industry experts.
- Education about food safety and production systems at ground level should be made easily accessible.
- Implementation of the Health Star Rating system which can assist consumers to conveniently compare the nutrition of products they purchase as well as providing clear food labels.

## Conclusion

The perception about public healthcare is oriented on hospitals, clinics, pharmaceutical companies, insurance companies and doctors for improvement of the well-being of the public. That has created huge health disparities between those who can afford and those who cannot afford medical aid cover; hence, the health disparities thereof. Public health disparities are thus caused by the classes in terms of access to healthcare services. The needs of the poor in relation to accessing health facilities are compromised due to a gap between the lower classes, middle classes and upper class when it comes to public health services. Accordingly, there are people who afford medical health services but are faced with the challenges of food choices for the sake of their health. Then it shows that there is a concern about the kind of food produced for the purpose healthcare. Then a conclusion can be made that, there is a concern about the modern food production system irrespective of one being able to afford medical services or not. From the paper’s discourse, it can be learned that perceptions about the safety of food products cannot be guaranteed to take a certain shape nor to be accepted as being influenced by a single occurrence. Whilst, on the other hand, there is an escalating cost in respect of healthcare that is worrying due life expectancy deteriorating and people seeming to be left to live with chronic diseases.

## Data Availability

Not applicable.

## Abbreviations

GHS: General Household Survey
GMF: Genetically Modified Foods
Stats SA: Statistics South Africa
WHO: World Health Organization
